# The Book World of Medicine and Science

**Published:** 1897-12-04

**Authors:** 


### The Book World of Medicine and Science.

Ringworm and Alopecia Areata, their Pathology, Diagnosis, and Treatment. By H. Aldersmith, M.B.Lond., F.R.C.S., Medical Officer, Christ's Hospital, London. (Fourth edition. London: H. K. Lewis. 1897. Price 10s. 6d.)

So large a book on what, so far as the text books go, is so small a subject may make some people wonder where specialism is to end. But in fact the subject dealt with becomes by no means a small one, wherever numbers of children have to live together, while the increase in our knowledge concerning it which has taken place during recent years makes the present a most appropriate time for the issue of a new and re-written edition of this well-known work. A good description is given as to the pathology of the diseases dealt with. The plurality of the fungi engaged is insisted on, and is well illustrated by a series of excellent plates. A very interesting question is discussed which has a bearing on many infectious diseases besides ringworms, namely, What is it that makes some individuals so much more subject to the disease than others? and it is pointed out that when puberty is reached, at from fourteen to fifteen years of age, ringworm of the scalp becomes much more manageable, and generally soon gets well spontaneously, even when it has existed for years. Complete directions are given as to the examination of the head, and it is shown how minute and careful the practitioner must be before he ventures to say that a patient has been cured. Under the head of treatment cases of tinea tonsurans may, according to the author, be divided into three classes: (1) Those in which only one or a few small places exist. (2) Extensive forms. (3) Disseminated ringworm. For the first various parasiticides are available; an alphabetical list of the most useful of these is given, and the indications for using the one one or the other are pointed out. In all such cases, however, it is well that the hair should be cut to about an inch in length, and that some parasiticide should be applied to the whole of the scalp as soon as possible to prevent the disease spreading. For this purpose the author's favourite applications are either an ointment of sulphur and salicylic acid, or one of sulphur, ammoniated mercury, and creasote. These ointments should be freely rubbed into the roots of the hair once a day directly after washing the head. In later stages carbolic glycerine (1 in 8) may be used. Cases of extensive tinea tonsurans are often very difficult to deal with, and many months may be required for their cure, notwithstanding that to the parents they may seem nearly well. The applications spoken of with most confidence are oleate of mercury; a lotion of boric acid, ether, and spirit of wine; and "gas water." Preference is given to the lotion or the gas water, as by this means the disease is sometimes completely cured, while this is rare with ointments or oleates, certain patches or stumps being left which require treatment later on with croton oil. A warning must be given about the offensive smell of gas water. This is so great that it can hardly be used in a small house. The author says, "I have known the neighbours send the sanitary inspector in to inspect the drains when a child was under this treatment. Before ordering it parents ought to realise what a nuisance the smell may be."

Finally, in cases of disseminated ringworm, especially that which may be described as the "black-dot" form, needling with croton oil is the treatment recommended. It must be pointed out, however, that this treatment is painful, that in its course a considerable amount of inflammation must necessarily be set up, and that as the success of the treatment depends largely on setting up enough and not too much of this inflammation considerable experience in its details is desirable. "Sometimes even when the case is being properly attended to the parents imagine the doctor is making the child much worse; they get alarmed, seek other advice and may be told that their former medical attendant ha been greatly over treating the case, and has been using too strong remedies." Still, when carefully used, Dr Aldersmith maintains that it is a most effectual treatment, and is applicable to many forms of the disease. This book is likely to be of great service to those who have these cases to deal with. No royal way to health, however, is pointed out; for the teaching of the book throughout is minute and painstaking care.

The Elements of Hypnotism. By R. H. Vincent. (London: Kegan Paul, Trench, and Co. 1897. Vol. LXXIV. International Scientific Series. Second Edition, 8vo, pp. 272. Illustrations 17. Price 5s.)

Mr. Vincent, in preparing this second edition of his well-known monograph, has written an entirely new chapter on the physiology of hypnotism. As the author candidly observes, on this subject his theories often fail to harmonise with the conclusicns of many leading authorities. In opposition to Von Ziehern and other authors, Mr. Vincent expresses his belief in the possibility of unconscious psychical action, and at the same time uses the word "psychical" to connote the non-physical aspects of nervous processes. Mr. Vincent, in dealing with the varieties of action, discards the terms "reflex" and "automatic," preferring those of "inaptic" and "aptic." Inaptic actions are those usually spoken of as reflex; aptic actions are those due to "unconscious psychical" processes. The author's theory of hypnosis practically is that, in the hypnotic state, by reason of abnormally induced inhibition of consciousness, acts usually attended by conscious processes are performed as if they were merely "aptic." That "thought is ultimately as much a physical fact as the process of secretion in a gland," is not the "fact" that the author states it is (page 199). It is a philosophic assumption, by its very nature incapable of proof, and its proposition implies at least neglect of what Huxley called the "irrefragible position of Berkeley." The chapters dealing with the genesis, induction, and phenomena of hypnosis give excellent and lucid accounts of their respective subjects; the remarks on the dangers of hypnotism are forcible, and the suggestions for its use moderate and restrained. But, although doubtless "functional" murmurs may be modified during hypnosis owing to variations of respiration and posture, we cannot accept, without reservation, the statement that the murmur of mitral stenosis may disappear on the induction of the hypnotic state. The author approaches the subject of Dr. Luys' experiments in a spirit of healthy scepticism; but we should have been glad to find some discussion of the phenomena of "double consciousness" and allied states, which surely have much in common with the condition of hypnosis. Mr. Vincent is certainly to be congratulated on the lucid and comprehensive book he has written, no less than on the pitfalls he has avoided in dealing with a fascinating but very difficult subject.

